# Dengue infection in kidney transplant recipients: clinical course and its impact on renal function

**DOI:** 10.1590/2175-8239-JBN-2021-0127

**Published:** 2021-09-24

**Authors:** Claudia Ribeiro, Sylvia Aparecida Dias Turani, Silvana Maria Carvalho Miranda, Pedro Augusto Macedo de Souza, Maria Goretti Moreira Guimarães Penido

**Affiliations:** 1 Santa Casa de Belo Horizonte, Centro de Nefrologia, Belo Horizonte, MG, Brasil.; 2 Universidade Federal de Minas Gerais, Faculdade de Medicina, Departamento de Pediatria, Belo Horizonte, MG, Brasil.

**Keywords:** Dengue Virus, Kidney Transplantation, Immunosuppression, Primary Graft Dysfunction, Vírus da dengue, Transplante de Rim, Imunossupressão, Disfunção Primária do Enxerto

## Abstract

**Introduction:**

Kidney transplant recipients (KTR) are at increased risk for dengue virus (DENV) infection. The aim of this study was to outline the clinical presentation and laboratory profile of DENV infection in KTR and its impact on renal function.

**Methods:**

This was a retrospective study of KTR diagnosed with DENV infection. Adult patients who visited Santa Casa de Belo Horizonte Nephrology Center between April and September 2019 were included. Patients who did not sign the Informed Consent were excluded. Data were collected from the database and medical records. The study was approved by the local Institutional Ethics Committee and the Informed Consent was obtained.

**Results:**

Nineteen KTR were evaluated. The main signs and symptoms were myalgia, headache/retro-orbital pain, fever, and gastrointestinal symptoms. Two patients had acute cholecystitis without calculus, three experienced pleural and/or pericardial effusion, and one developed acute myocarditis. All patients were under immunosuppression with prednisone, tacrolimus, and mycophenolate, and most were not receiving induction therapy. Temporary suspension/reduction of immunosuppression was required in 58% of patients and leukopenia was the most common reason. Thrombocytopenia was common and 58% of patients developed acute kidney injury. All patients recovered renal function.

**Conclusions:**

DENV infection in KTR patients seems to follow a similar course as in the general population. Although there was no control group, we suspect that immunosuppression, preexisting kidney disease or type of donor was not a determining factor in most patients. Transient renal dysfunction was common but reversible. No patient experienced death or graft loss.

## Resumo

**Introdução:**

Receptores de transplante renal (RTR) apresentam maior risco de infecção pelo vírus da dengue (DENV). O objetivo deste estudo foi delinear apresentação clínica e perfil laboratorial da infecção por DENV em RTR e seu impacto na função renal.

**Métodos:**

Este foi um estudo retrospectivo de RTR diagnosticados com infecção por DENV. Incluiu-se pacientes adultos que estiveram no Centro Nefrológico da Santa Casa de Belo Horizonte entre Abril/Setembro, 2019. Foram excluídos pacientes que não assinaram o Termo de Consentimento Livre e Esclarecido. Dados foram coletados do banco de dados e registros médicos. O estudo foi aprovado pelo Comitê de Ética Institucional local e obteve-se o Consentimento Livre e Esclarecido.

**Resultados:**

Avaliou-se dezenove RTR. Principais sinais e sintomas foram mialgia, cefaleia/dor retro-orbital, febre, sintomas gastrointestinais. Dois pacientes apresentaram colecistite aguda sem cálculo, três sofreram derrame pleural e/ou pericárdico, um desenvolveu miocardite aguda. Todos os pacientes estavam sob imunossupressão com prednisona, tacrolimus, micofenolato. A maioria não estava recebendo terapia de indução. Suspensão/redução temporária da imunossupressão foi necessária em 58% dos pacientes e leucopenia foi a razão mais comum. Trombocitopenia foi comum e 58% dos pacientes desenvolveram lesão renal aguda. Todos recuperaram a função renal.

**Conclusões:**

A infecção por DENV em pacientes RTR parece seguir um curso semelhante ao da população em geral. Embora não houvesse grupo controle, suspeitamos que imunossupressão, doença renal preexistente ou tipo de doador não foram fator determinante na maioria dos pacientes. Disfunção renal transitória foi comum, mas reversível. Nenhum paciente veio ao óbito ou sofreu perda do enxerto.

## Introduction

Dengue virus (DENV) infection is an arthropod-borne disease caused by an RNA virus of the Flaviviridae family that is transmitted primarily by the mosquito *Aedes aegypti*.^1^ There are four serologically distinct dengue viruses: DENV-1, DENV-2, DENV-3 and DENV-4. Infection with one serotype does not confer immunity to other serotypes.^2^ DENV infection can occur as an endemic disease or as epidemic outbreaks.^3^ It is endemic in many tropical and sub-tropical countries, such as Brazil, the Caribbean, and Southeast Asian countries, and causes an enormous economic burden, especially for the health sector.^3^ Furthermore, rapid urbanization with overpopulation in tropical and subtropical countries favors the association of dengue epidemics and major lifestyle changes with the onset of diabetes, high blood pressure and, consequently, chronic kidney disease (CKD).^4^^,^ 5

Annually, approximately 390 million people become infected with DENV, and 3.9 million live in countries where it is endemic.^6^^,^ 7 According to the Brazilian Ministry of Health (BMH), 971,136 dengue cases were registered from January to November 2020, with an incidence rate of 462.1 cases per 100 thousand population and 528 deaths in the country.^8^

The illness can be asymptomatic or symptomatic, and common symptoms are fever, myalgia, arthralgia, headache, skin rash, and retro-orbital pain. It can cause a systemic and dynamic disease that varies from mild to severe form and may lead to death.^9^ Dengue can be divided into two main categories: undifferentiated dengue (with or without warning signs) and severe dengue. This classification of dengue fever was designed to help healthcare workers and minimize the risk of contracting severe dengue fever.^10^

Recently, there has been an increase in kidney transplants in Brazil.^12^ Kidney transplant recipients (KTR) living in endemic areas are at higher risk of DENV infection, and this vulnerable group of immunosuppressed patients can develop a more severe disease.^5^ However, previous studies suggest that dengue fever is mild in KTR and the disease does not affect allograft function.^2^^,^ 3 Although severe morbidity (graft failure that needs dialysis and graft nephrectomy)^3^ and mortality^11^ had been reported, severe dengue infection is thought to be due to immune-mediated mechanisms and may not occur in transplant recipients who have a muted immune response.^2^^,^ 3 ^,^ 7

Unfortunately, there are few studies that address this issue in the literature. The aim of this study was to outline the clinical presentation and laboratory profile of DENV infection in KTR and the impact of the disease on kidney function.

## Materials and Methods

### Study design

This was a retrospective study of KTR that were diagnosed with DENV infection.

### Patients

Adult KTR diagnosed with DENV infection confirmed by laboratory test at Santa Casa de Belo Horizonte Nephrology Center from April to September 2019 were included. This hospital has a comprehensive kidney transplant program where patients receive free transplant surgery and medications for life. Patients are followed up regularly and referred for evaluation in the event of graft dysfunction or serious illness. Patients who do not signed the Informed consent were excluded.

## Methods

Data were collected from the database of the Santa Casa de Belo Horizonte Nephrology Center, where patient data are registered, and from patients' medical records. The variables collected were: demographic and clinical data (sex, age, length of hospital stay, dengue signs and symptoms and clinical stage), transplant data (donor type, immunosuppression, induction), and laboratory data (serological diagnosis, biochemical kidney tests, blood tests, kidney function test). The CKD-EPI formula was used for the glomerular filtration rate calculation.^13^

A dengue case was defined according to BMH 2016, Center for Disease Control and Prevention (CDC) 2015 and World Health Organization (WHO) 2009 as: a compatible clinical disease with either positive serology (IgM dengue antibody) and/or positive NS1 antigen, during the study period.^9^^,^ 14 ^-^ 16

Dengue patients were classified in Group A - dengue without warning signs, no special condition, no social risk, and no comorbidities; Group B - dengue without warning signs, with special condition, or with social risk and comorbidity; Group C - dengue with warning signs and no severe signs: severe abdominal pain (referred to or on palpation) and continuous, persistent vomiting, fluid accumulation (ascites, pleural effusion, pericardial effusion), postural hypotension and/or lipothymia, hepatomegaly greater than 2 cm below the costal margin, mucosal bleeding, lethargy and/or irritability, and progressive increase in hematocrit; Group D - severe dengue: severe plasma extravasation causing shock recognizable by tachycardia, weak and thready pulse, slow capillary refill (>2 seconds), convergent blood pressure (<20 mmHg), tachypnea, oliguria (<1.5 mL/kg/h), hypotension and cyanosis (late stage of shock), accumulation of fluids with respiratory failure, severe bleeding, and severe organ impairment.^10^

Standard treatment protocol for dengue was followed according to BMH (2016)^9^,, CDC (2015)^15^ and WHO (2009)^16^ Classic dengue is characterized by seven-day fever and at least two nonspecific signs and symptoms (headache, malaise, retro-orbital pain, exanthema, myalgia, and arthralgia). Hemorrhagic dengue is characterized by increased vascular permeability leading to bleeding diathesis or disseminated intravascular coagulation, with at least one of the following signs or symptoms: hemorrhagic manifestations, hemoconcentration due to capillary leak, hypoproteinemia, and pleural effusion or ascites. Dengue shock syndrome was considered for all severe cases not fulfilling the hemorrhagic dengue criteria and the classic dengue classification, with one of the following clinical findings: several changes in the nervous system, cardio-respiratory dysfunction, liver failure, thrombocytopenia equal to or less than 20000/mm^3^, digestive bleeding, pleural effusions, global leukocyte count equal to or less than 1000/m^3^, suspected case of dengue evolving to death.

The differential diagnosis for Zika and Chikungunya was made by laboratory tests (positive IgM serology and/or positive NS-1). However, only one third of the patients was tested. The KDIGO criteria was used to classify acute renal failure.^17^

The immunosuppressive drugs taken by patients at the time of dengue presentation included: cyclosporine (CSA), azathioprine (AZA), tacrolimus (TAC), or mycophenolate mofetil (MMF) in combination with low- or high-dose oral steroids.

Leukopenia was defined as leukocyte count below 3500/mm^3^ and thrombocytopenia was defined as platelet count below 150.000/mm^3^. Graft dysfunction was defined as an absolute increase in serum creatinine by ≥ 0.5mg/dL.

### Statistical analysis

All data were entered and analyzed in JASP 0.14.1 (University of Amsterdam). Qualitative variables were expressed in absolute frequencies and percentages. Categorical and continuous data were described as absolute numbers and relative frequencies and as mean and median with standard deviation (SD) and interquartile ranges (IQRs), respectively. The t-test was used for comparison of creatinine before, during, and after hospitalization. A two-sided p < 0.05 was defined as significant.

### Ethical Aspects

The study was approved by the Institutional Ethics committee review board (nº 3.806.077) and was conducted in accordance with the ethical standards established in the 1964 Declaration of Helsinki. Informed consent was obtained from all patients.

## Results

Nineteen KTR diagnosed with dengue were evaluated. Demographic, hospitalization, and transplant characteristics are described in Table 1. Most patients were admitted 3 days after showing initial symptoms and remained hospitalized for eleven days. The main signs and symptoms were myalgia, headache/retro-orbital pain, and fever (Table 1). Gastrointestinal symptoms were also quite common, mainly manifested by nausea and vomiting, abdominal pain, and diarrhea (Table 1). Two patients presented clinical and ultrasonographic signs consistent with acute cholecystitis without calculus, which improved with resolution of the viral infection. Three patients experienced pleural and/or pericardial effusion and one developed acute myocarditis with thoracic pain and transient elevation of myocardial necrosis markers (Table 1).

**Table 1:** Evolution of creatinine values (mg/dL) during and after the DENV infection in the kidney transp lant patients (n = 19).

Age in years (median: max - min)	43 (17 - 70)
Sex	
Female (n/%)	14/74
Male (n/%)	5/26
Donor type (n/%)	
Living	8/42
Deceased	11/58
Induction (n/%)	
Yes	8/42
No	11/58
Time since transplant (months - mean ± SD)	72 ± 57.5
ISS at the time of admission for DENV (n/%)	
PRED / TAC / MFS	12/63
PRED / TAC / AZA	3/16
PRED / TAC / SRL	3/16
PRED / TAC / EVR	1/5
ISS reduction (n/%)	
Yes	11/58
TAC	1/5
MFS	7/37
AZA	2/11
SRL	1/5
No	8/42
Length of hospital stay in days (mean ± SD)	11 ± 12
Reason for hospitalization (n/%)	
Leukopenia	10/53
Unknown	9/4
Dengue clinical staging according to BMH 20169 (n/%)	
Group B	4/21
Group C	13/68
Group D	2/11

PRED: Prednisone, TAC: Tacrolimus, MFS: Mycophenolate sodium, SRL: Sirolimus, AZA: Azathioprine, TG: Thymoglobulin, BSX: Basiliximab, EVR: Everolimus, SD: Standard deviation, BMH 2016: Brazil Ministry of Health^9^

According to the risk classification,^9^ most patients were classified as C group. Two patients required intensive care: one due to diabetic ketoacidosis and the other due to hemorrhagic shock resulting from digestive tract bleeding. None of the patients died or suffered graft loss.

All patients were under a triple immunosuppression regimen and the most common drug combination used was with prednisone, tacrolimus and mycophenolate (Table 1).

Most patients did not receive induction therapy, and there were no differences between the disease severity and the use of different immunosuppression regimens or the intensity of laboratory alterations (Table 1). Temporary suspension or reduction of immunosuppression was required in 58% of patients with leukopenia being the most common reason (Table 2).

**Table 2:** Laboratorial and clinical characteristics of kidney transplant patients with dengue (n = 19)

Leukocyte count (mean ± SD; per mm3)	3,341 ± 1574,8
Lowest leukocyte count	1,100
Highest leukocyte count	6,000
Platelet count (mean ± SD; per mm3)	80,105 ± 59342,5
Lowest platelet count	16,000
Highest platelet count	193,000
Hemoglobin count (mean ± SD; g/dL)	10.9 ± 3.25
Lowest hemoglobin count	4.8
Highest hemoglobin count	16.0
Baseline serum creatinine (mean ± SD; mg/dL)	1.74 ± 0.617
Lowest creatinine	0.87
Highest creatinine	2.87
Rise in serum creatinine from baseline (mean ± SD; mg/dL)	2.45 ± 1.35
Lowest creatinine	0.98
Highest creatinine	5.96
Final creatinine (mean ± SD; mg/dL)	1.71 ± 0.75
Lowest final creatinine	0.94
Highest final creatinine	3.80
Clinical features (n/%)	
Myalgia	12/63
Headache	9/47
Fever	8/42
Nauseas and vomiting	8/42
Abdominal pain	5/26
Diarrhea	4/21
Hyporexia	4/21
Retro-orbital pain	3/16
Prostration	3/16
Cough	3/16
Digestive bleeding	2/10.5
Chest pain	2/10.5
Dyspnea	2/10.5
Acalculous cholecystitis	2/10.5
Pericardial effusion	2/10.5
Arthralgia	1/5.2
Skin rash	1/5.2
Postural hypotension	1/5.2
Pruritus	1/5.2
Myocarditis	1/5.2
Arterial hypertension	1/5.2
AKI	1/5.2
Coryza	1/5.2

AKI: Acute kidney injury

Thrombocytopenia was the most frequent laboratorial alteration, appearing in 79.0% of the patients, and 47.3% experienced platelet counts below 50.000/mm^3^. Leukopenia was also frequent and in 21% of them it was less than 2000/mm^3^. Two patients had severe anemia and required blood transfusion due to digestive tract hemorrhage (Table 2).​

**Figure 1:**
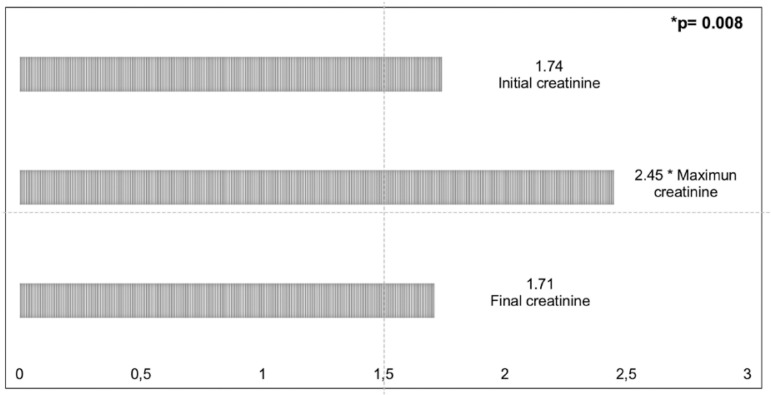
Evolution of creatinine values (mg/dL) during and after the DENV infection in the kidney transplant patients (n = 19).

Fifty-eight percent of patients developed AKI, but all recovered renal function (Figure 1). During DENV infection, the mean creatinine was 1.71 mg/dL and rose significantly, reaching levels up to 2.45 mg/dL (p = 0.008) and returning to baseline levels at the end of hospitalization (Cr 1.73 mg/dL; p = 0.80).

## Discussion

Dengue fever is a mosquito-borne viral disease that is very common in Latin America, especially in Brazil, where it is endemic and epidemic. Data from the Brazilian Ministry of Health (MS) show that 1,544,987 cases of the disease were reported (735.2 cases/100 thousand population) in 2019. The states of Minas Gerais, São Paulo and Goiás account for 67.9% of the probable cases in the country.^18^

​The clinical profile in healthy subjects ranges from asymptomatic to nonspecific symptoms such as cephalea, arthralgia, fever, weakness, myalgia, lower-back pain, retro-orbital pain and malaise to more severe conditions such as leukopenia, thrombocytopenia, bleeding and dysfunction of other organs and systems.^2^^,^ 3 ^,^ 5 ^,^ 7 ^,^ 14 KTR living in or who travel to endemic areas have higher risk of acquiring the disease in its varied spectrum of manifestations.^19^

Data about the presentation and outcome of the DENV infection in KTR are limited. The question whether this infection takes a more benign or severe course in transplant recipients than in the general population remains unknown. The use of immunosuppressive drugs (ISS) by KTR could result in an insufficient immune response to DENV infection with more prolonged viremia, allowing a more severe clinical presentation of the disease. However, this is not yet an evidence.^14^

In the present study, we described 19 dengue cases in KTR from a single center located at an endemic area over a period of 6 months. Most patients were women and received a deceased donor graft, according to Fernandes et al (2017).^19^ However, Nasin et al (2013)^2^, Costa et al (2015)^3^ and Weerakkody et al (2017)^7^ found that most of their patients were male.^2^^,^ 3 ^,^ 7 The mean age of patients from the study by Nasim et al. (2013)^2^ was 28 years^2^ whereas the median age of our study patients was 43 years, which is close to the mean age reported in a systematic review by Weerakkody et al 2017)^7^ of 38.9 ± 13.8 years.

As described by (2013)^2^, KTR with dengue tend to have a longer disease duration compared with the general population, and consequently, a prolonged hospital stay.The authors reported a mean length of hospital stay of 13 ± 12 days compared to 4 ±2.3 days in the general population, which may be attributed to comorbidities in transplant recipients.^1^^,^ 2 In our study, the mean length of stay was 11 days, which was similar to that reported by Subbiah et al (2018)^15^ (9 days).The main reason for hospitalization in the present study was leukopenia (Table 1), but other studies have shown severe thrombocytopenia and bleeding as the principal cause.^2^^,^ 5

Most of our patients were receiving PRED, TAC, and MFS for immunosuppression at the time of hospital admission due to dengue (Table 1), which was similar to what Fernandes et al. (2017)^19^ reported.Nasim et al. (2013)^2^ used cyclosporine (CSA), azathioprine (AZA) and PRED as ISS in the majority of their patients.

Reduction of ISS was necessary in 11 of our patients, especially in those using antiproliferative drugs, due to cytopenia. Fernandes et al. (2017)^19^ described the need for temporary reduction or suspension of ISS in their 11 KTR with DENV infection, especially of the drug MIF, also due to cytopenias.According to BMH (2016)^9^,most of our patients (68%) had Group C dengue (Table 1).

Regarding laboratory alterations, our patients frequently showed leukopenia and thrombocytopenia, but without an increase of hematocrit, as described in the general population. Some patients already had polycythemia before viral infection, others had anemia mainly due to bleeding. As commented by Nasim et al. (2013)^2^, KTR presenting with thrombocytopenia in endemic areas, should be highly suspicion for dengue even if afebrile.They found severe thrombocytopenia in 44% of their patients^2^ compared to 60% in the general population reported by Riaz et al. (2009)^20^, while there were no cases in the series by Prasad et al. (2012)^12^ It seems that KTR have less severe thrombocytopenia. Fernandes et al. (2017)^19^reported thrombocytopenia in most of their cases, with only 33.6% of severe thrombocytopenia. The mean time of thrombocytopenia was 9 days, which is higher than the general population.^19^ This fact was also seen by Nasim et al. (2013)^2^ with mean thrombocytopenia duration of 11 ± 9 days, compared with 3.6 ± 1.6 days in the general population.^21^ Thrombocytopenia with minimum platelet count ranging from 15,300/mm^3^ to 99,000/mm^3^ was described in 70% of the patients by Costa et al. (2015)^3^.

Prolonged thrombocytopenia has been observed in transplant recipients with dengue in the general population.^20^ Immunocompromised patients might have slow viral clearance causing continued platelet destruction.^22^ Studies have suggested that TAC may cause prolonged thrombocytopenia. However, the role of immunosuppressants in thrombocytopenia in transplant recipients with dengue needs further study.^2^^,^ 14 ^,^ 19

As mentioned earlier, leukopenia occurred frequently in our patients (Table 2), similar as described by Subbiah et al. (2018)^15^ and Wiwanitkit (2010)^23^This is due to the cumulative effect of ISS treatment and viral infection. Arun Thomas et al. (2019)^5^ showed that leukocyte count was lowest in KTR compared to CKD patients and the control group, probably due to bone marrow suppression by MMF and AZA used for ISS. This might also be another reason why it takes longer time for platelet count to normalize in KTR.

In the present study patients developed mostly common symptoms and signs, often described in the general population, with emphasis on gastrointestinal (nauseas, vomiting, abdominal pain, diarrhea, hyporexia) and respiratory manifestation (cough, chest pain, dyspnea). It should be noted that fever was not as prevalent as in the general population, affecting less than half of the sample (Table 2). Dengue in KTR has been described by Renaud et al. (2007)^22^ (6 patients in Singapore)^22^ and Azevedo et al. (2007)^24^ (27 patients in Brazil) as a disease with good course. However, Wiwanitkit (2010)^23^ from Iran and Prasad et al. (2012)^12^ from India described a more severe course. Subbiah et al. (2018)^15^, Fernandes et al. (2017)^19^, and Costa et al. (2015)^3^ reported that fever was present in the absolute majority their allograft recipients with dengue.^3^^,^ 14 ^,^ 19 The afebrile dengue has been attributed to the regular steroid intake in this group of patients.^25^ Also, the absence of classic DENV infection symptoms such as fever in elderly patients is due to age-related decline in immune functions and poor cytokine response.^26^^,^ 27 Nasim et al. (2013)^2^ reported that 20% of their patients did not have fever and the duration of fever in those patients who had it was the same as in dengue in the general population.^2^ This fact has been observed mostly in patients using larger ISS doses. Fernandes et al. (2017)^19^ found that patients on high-dose steroids had significantly less fever.^19^ According to Teixeira et al. (2002)^28^, transplant patients may not present a febrile response because of the ISS therapy, especially if taking steroids.^28^

Arthralgia is commonly seen in dengue patients, but it was not observed in our study (Table 2). Subbiah et al. (2018)^15^ and Prasad et al. (2012)^12^ in India, and Azevedo et al. (2007)^24^ in Brazil also did not observed arthralgia in their patients.^11^^,^ 14 ^,^ 24 This is probably due to steroid usage among transplant recipients.

In 2007, Gulati and Maheshwari^29^ reported dengue atypical symptoms affecting the nervous, gastrointestinal, cardiac, and renal systems.Two of our KTR developed atypical cholecystitis, characterized by pain in the right hypochondrium, fever, positive Murphy's sign, altered liver enzymes, and thickening of the gallbladder wall without stones. This is not a common clinical condition in dengue and the pathophysiology is not yet well known. The prolonged fasting, spasm of the duodenal papilla, endotoxemia, microangiopathy, and ischemia-reperfusion injury have been suggested as possible causes of cholestasis and increased viscosity of bile secretion.^29^ In dengue hemorrhagic fever, the direct viral entry, increased vascular permeability causing leakage, and serous effusion of protein content could cause thickening of the gallbladder wall.^29^^,^ 30 Similarly, cardiac involvement is rare in classic dengue and may manifest as myocarditis and pericarditis, and heart failure, including electrocardiographic changes.^31^ In our study, one patient was admitted to the Coronary Care Unit under suspicion of acute myocardial infarction, but viral myocarditis was observed later. Miranda et al described a series of 81 patients diagnosed with dengue in the general population. The authors found 12 cases positive for at least one cardiac marker and 8 patients had a symptom suggestive of cardiac involvement (acute heart failure, chest pain, and severe hypotension).^32^

In 2011, the WHO announced the term "expanded dengue syndrome" (EDS) to refer to cases that do not fall under either dengue hemorrhagic fever or dengue shock syndrome, and have unusual manifestations in other organs such as the cardiovascular, nervous, kidney, intestinal, and the hematologic systems.^33^ Currently, reports of under-documented and rare manifestations with severe organ involvement are accumulating. The EDS category helps to establish the diagnosis and prompt treatment of dengue with unusual manifestations.

Previous studies suggested that dengue had a mild impact on KTR, with good recovery, low death risk, and minimal graft function impact.^2^^,^ 23 ^,^ 24 Other publications on KTR with DENV infection report symptoms and signs similar to those in the general population, but with higher occurrence of warning manifestations.^34^ According to Renaud et al. (2007)^22^ and Azevedo et al. (2007)^24^, kidney transplant patients treated with multiple ISS should be less likely to develop the severe form of DENV infection.^22^^,^ 24 The latter is thought to occur after secondary infection due to the phenomenon known as antibody dependent enhancement (ADE) response.^33^^,^ 34 It could be argued that they are less susceptible because the humoral immune response is suppressed in KTR.

The Subbiah et al 2018)^15^ group reported two patients with DENV infection in the post-transplant period that had spontaneous recovery without any complications.^14^ However, (2015)^3^ and Prasad et al (2012)^12^ showed that DENV infection in the post-transplant period was more serious in their patients, although none died.^3^^,^ 11 In agreement with the last two authors, Wiwanitkit (2010)^23^ commented that a considerable number of severe cases are reported in KTR.^23^

Weerakkody et al. (2017)^7^ compared a total of 168 KTR with the general population, and they have concluded that KTR had significantly less common dengue clinical manifestations such as fever, myalgia, and arthralgia.The authors concluded that the physical and laboratory findings in those patients do not differ from those in the general population and they had significantly more clinical complications and death.

According to Nasim et al. (2013)^2^, mortality in their patients occurred mostly in those who had concomitant sepsis, and therefore cannot be attributed to DENV infection alone. The authors suggested that DENV infection should be considered a co-infection that may have contributed to the overall mortality in the study.^2^ They described a mild disease with good recovery in the majority of the patients. Only one-quarter of those who developed the severe form of the disease had primary DENV infection and three-quarters had secondary infection, indicating that ISS in KTR does not confer protection against antibody-mediated enhancement seen in secondary infection.^2^ Fernandes et al. (2017)^19^ from Brazil described 11 kidney allograft recipients with DENV infection and confirmed the benign nature of the disease in KTR, without mortality. Although 81% of the patients had thrombocytopenia, only 33% had severe thrombocytopenia and the infection tended to follow the usual course of the disease.

In our study, 58% of patients developed AKI, but 100% of them completely recovered renal function (Figure 1). A systematic review of the literature on DENV infection in KTR showed graft dysfunction in almost 60%,^7^ in agreement with our study. The authors also found an average creatinine increase of 61.7% during the course of the disease. Mortality was 8.9%, which was higher than that of the normal population (0.062%), but the majority recovered.^7^ Nasim et al. (2013)^2^ reported that more than half of their patients had graft dysfunction, but most returned to pre-DENV renal function levels within two weeks. Those whose graft function was already significantly compromised before dengue were more likely to have persistent graft dysfunction. They concluded that DENV infection alone does not cause significant graft dysfunction in the absence of other factors in KTR.^2^

All patients of the Subbiah et al. (2018)^15^ study recovered normal renal function by the time of recovery from DENV infection. The authors suggested that renal dysfunction is most likely due to the febrile illness, associated dehydration, and other infection-related factors rather than a direct viral cytopathic effect.^14^ similarly, 80% of the patients of the Costa et al. (2015)^3^ study in Brazil showed slight worsening during the DENV infection, but returned to basal creatinine levels after infection recovery. Three patients needed RRT, two had graft loss, and one needed dialysis for three weeks and had full recovery of graft function.^3^ Azevedo et al. (2007)^24^also showed a transitory dysfunction of the kidney graft during DENV infection. Fernandes et al. (2017)^19^ found an increase of mean creatinine from 1.35 to 2.5 mg/dL in the DENV infectious period, which are values very close to those found in our study (Figure 1). The authors informed that the grafts were not damaged at medium or long term and recovery of all patients was satisfactory with mean creatinine of 1.1 mg/dL in the post-infectious period.^19^ Probably, this behavior was not due to the direct action of the virus in the kidney parenchyma, but due to factors associated with dehydration/hypovolemia caused by capillary leakage, vomiting, or bleeding.^35^ ​We observed similar kidney function behavior in our patients during the hospitalization period with significant and transient increase of serum creatinine and return to baseline levels. None of patients progressed to death or graft loss.

This study had some limitations and potential biases. This was a retrospective case series with data collected from medical records. It was not possible to assess the impact on disease morbidity and mortality in these patients because of the lack of serological surveys and routine serological screenings in asymptomatic patients. The strength of our study lies in the fact that the majority of our patients live in endemic DENV regions allowing the recording of their actual clinical evolution. Although this was a retrospective study, our case series stands out because of the severity of the cases. The majority of the patients were classified as a group C and required hospitalization because of the warning signs.^8^^,^ 9 Few studies had described such a large number of cases in such a short period of time, a total of nineteen cases in only six months. In addition, we reported two cases with acute acalculous cholecystitis and myocarditis (Table 2).

## Conclusions

​Dengue is a human viral mosquito-borne infection very important worldwide, which should be included in the differential diagnosis of KTR with fever. According with our results, dengue in KTR seems to have a course similar to the general population, although we did not have a control group. Some conclusions were not possible from these data given the lack of a comparable group and lack of comparison for the ISS and type of donor factors. Therefore, we conclude that the immunosuppression, preexisting kidney disease, or type of donor did not define the outcome in most patients.

We believe that special attention should be paid to patients whose signs and symptoms differ from those of classic DENV fever, as they may develop severe organic commitment, resulting in acute graft dysfunction. The main clinical signs and symptoms in our patients were myalgia, headache, fever, nauseas and vomiting, abdominal pain, diarrhea, and hyporexia. Compared with the general population, the frequency of signs and symptoms was quite similar, with the exception of arthralgia and retro-orbital pain, which were less common. Laboratory findings were leukopenia, thrombocytopenia and anemia, which are commonly associated with DENV infection. No patient experienced death or graft loss, with renal function improving at the end of hospitalization.
